# Two distinct Paleozoic metamorphic events in the Tarim-North China Collage

**DOI:** 10.1093/nsr/nwac133

**Published:** 2022-07-09

**Authors:** Jérémie Soldner

**Affiliations:** State Key Laboratory of Isotope Geochemistry, Guangzhou Institute of Geochemistry, Chinese Academy of Sciences, China; CAS Center for Excellence in Deep Earth Science, China

The Central Asian Orogenic Belt (CAOB) [1], which includes the Altaids in Central and Northern Asia [2], is the largest accretionary orogen formed during the subduction of the Paleo-Asian Ocean and the Panthalassa Ocean in the period of the Late Proterozoic to Late Paleozoic [3]. Closure of the Paleo-Asian Ocean is attributed to the northward drift of the North China and Tarim cratons leading to the massive shortening and oroclinal buckling of the CAOB accretionary system in the Permian [4]. This led Xiao and others [5] to define the CAOB as a supercollage formed of the northerly accretionary Kazakhstan and Mongolian Collages and southerly Tarim-North China Collage (TNCC).

High-grade metamorphic rocks from the central parts of the TNCC formed during the first Early Paleozoic orogenic cycle related to the assembly of Gondwana-derived microblocks within the Paleo-Asian Ocean [6,7]. Evidence of the later interaction of the TNCC with the southern CAOB is represented by high-grade metamorphic rocks from the northern Tarim and North China cratons (Fig. 1a). These rocks formed during the second orogenic cycle related to the final closure of the Paleo-Asian Ocean and agglomeration of Pangea [7]. As metamorphic *P–T* data record the geodynamic regimes [8], a review of age, temperature (*T*), pressure (*P*) and thermobaric ratio (*T/P*) of metamorphic rocks from the TNCC (Fig. 1b)and c, and Supplementary Table S1) is conducted to characterize Paleozoic tectono-metamorphic processes. The results highlight the fundamental differences between the two orogenic cycles (Fig. 1b)and c). The first cycle is mainly characterized by intermediate to high *T/P* metamorphism (Fig. 1c), represented by Ordovician to Early Devonian orogenic events that initiated by ∼460 Ma high-temperature eclogite-facies metamorphism in the Beishan Orogen and culminated with granulite-facies metamorphism of the Dunhuang block at ∼412 Ma. In comparison, the second cycle took place in the Late Paleozoic along the South Tianshan and Solonker segments of the Paleo-Asian suture with ages between 325 and 240 Ma. This cycle is characterized by the formation of pene-contemporaneous low and high *T/P* metamorphic rocks, exemplified by the low-temperature eclogite- and upper amphibolite–granulite-facies metamorphism, respectively (Fig. 1c).

**Figure 1.**
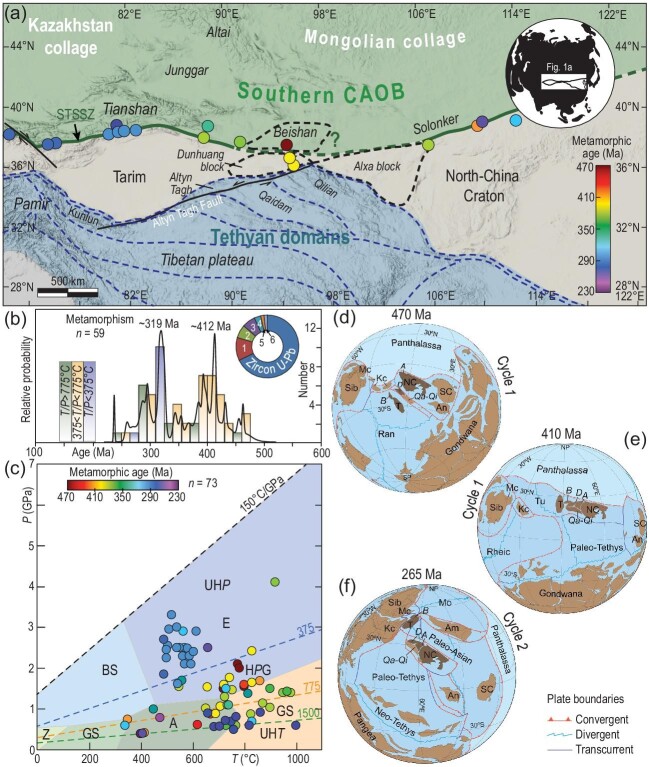
(a) Tectonic map of the southern CAOB and adjacent areas showing the distribution of high-grade metamorphic rocks (colored circles) in the Tarim-North China Collage (TNCC). STSSZ, South Tianshan-Solonker suture zone. (b) Relative probability density plots of representative metamorphic rocks from the southern CAOB (*n* = 59; Supplementary Table S1), with the rock's average *T/P* ratios calculated for each bin. The pie chart indicates the proportion of different geochronological methods used to calculate the relative probability density of metamorphic ages, with numbers 1–6 corresponding to geochronological methods listed in Supplementary Table S1. (c) Peak *P–T* diagrams of metamorphic rocks from TNCC (*n* = 73; Supplementary Table S1). Four representative thermal gradients and fields for the standard metamorphic facies are modified after Ref. [8]. Z, zeolite; BS, blueschist; E, eclogite; UH*P*, ultrahigh-pressure; GS, greenschist; A, amphibolite; H*P*G, high-pressure granulite metamorphism; G, granulite; UH*T*, ultrahigh-temperature metamorphism. (d)–(f) Position of the TNCC at ∼470 and ∼410 Ma (Cycle 1), and ∼260 Ma (Cycle 2) in the full-plate models of Refs [3,15], reconstructed using GPlate software [16]. Plate and terrane abbreviations: A, Alxa; Am, Amuria; An, Annamia; B, Beishan; D, Dunhuang; Kc, Kazakhstan collage; Mc, Mongolian collage; NC, North China; Qa, Qaidam; Qi, Qilian; SC, South China; Sib, Siberia; T, Tarim. Ocean abbreviation: Tu, Turkestan Ocean. Exterior and interior oceans are indicated in light and dark blue, respectively. Others: NP, North Pole; SP, South Pole.

The intermediate to high *T/P* metamorphic rocks from the Beishan-Dunhuang-Alxa-Tarim regions, characterized by both clockwise and anti-clockwise *P–T* evolution, suggest that the first orogenic cycle was related to thickening and thinning processes. Such an evolutionary tract may encompass subduction-to-collision events similar to those of the Cambrian East Gondwana margin [9] and supra-subduction thinning followed by thickening of thermally softened crust in active margins [6]. The absence of ultrahigh-pressure records together with recent paleogeographic reconstructions point to progressive closure of small interior oceanic basins between Gondwana-derived blocks [7], similar to the SE Asia archipelago [10].

The Late Paleozoic dual *T/P* metamorphism recorded in the South Tianshan-Solonker suture zone shares some similarities with classic peripheral-type ‘paired’ metamorphic belts [11]. However, ultrahigh-pressure metamorphic rocks from the South Tianshan display features of deep subduction and progressive exhumation of high-pressure sheets typical of the interior Alpine–Himalaya system [12], which is compatible with the subduction of a cold oceanic domain followed by the collision of the Tarim Craton with the Kazakhstan microcontinent [13].

The complied *P–T* data reveal a two-staged evolution of the TNCC: the Early to Middle Paleozoic high-temperature and medium-pressure supra-subduction metamorphic event corresponding to the construction of the collage (Fig. 1d) and the Late Paleozoic high-pressure subduction-to-collision metamorphic event related to the closure of the Paleo-Asian Ocean (Fig. 1e). The first orogenic cycle reflects tectonic switching supra-subduction mode [14], while the second one documents cold oceanic subduction in the east and transition from cold oceanic subduction to continental collision in the west. Based on this fundamental distinction, it can be argued that the tectono-metamorphic records of the TNCC reveals features of both peripheral–accretionary and interior–collisional orogens, which respectively correspond to two different and successive orogenic phases.

## Supplementary Material

nwac133_Supplemental_File
